# Rollout of a statewide Australian telestroke network including virtual reality training is associated with improved hyperacute stroke workflow metrics and thrombolysis rate

**DOI:** 10.3389/fstro.2024.1382608

**Published:** 2024-05-27

**Authors:** Carlos Garcia-Esperon, Steven Maltby, Ken Butcher, Md Golam Hasnain, Beng Lim Alvin Chew, William O'Brien, James W. Evans, Timothy Ang, Leon Edwards, Christopher Blair, Candice Delcourt, Mark W. Parsons, Ferdinand Miteff, Jason Dizon, David Lambkin, Daniel Barker, Murielle G. Kluge, John H. Wiggers, Christopher R. Levi, Neil J. Spratt, Frederick Rohan Walker, The Virtual Reality NSW Telestroke Group, Chris Oldmeadow

**Affiliations:** ^1^Department of Neurology, John Hunter Hospital, New Lambton Heights, NSW, Australia; ^2^Hunter Medical Research Institute, New Lambton Heights, NSW, Australia; ^3^Centre for Advanced Training Systems, The University of Newcastle, Callaghan, NSW, Australia; ^4^School of Biomedical Sciences and Pharmacy, College of Health, Medicine and Wellbeing, The University of Newcastle, Callaghan, NSW, Australia; ^5^School of Clinical Medicine, University of New South Wales, Sydney, NSW, Australia; ^6^Department of Neurosciences, Gosford Hospital, Gosford, NSW, Australia; ^7^Department of Neurology and Neurointervention, Royal Prince Alfred Hospital, Camperdown, NSW, Australia; ^8^Department of Neurology, Liverpool Hospital, Ingham Institute for Applied Medical Research, University of New South Wales South Western Sydney Clinical School, Liverpool, NSW, Australia; ^9^The George Institute for Global Health, Faculty of Medicine, University of New South Wales, Sydney, NSW, Australia; ^10^Department of Clinical Medicine, Faculty of Medicine, Health and Human Sciences, Macquarie University, Macquarie Park, NSW, Australia; ^11^School of Medicine and Public Health, College of Health, Medicine and Wellbeing, The University of Newcastle, Callaghan, NSW, Australia; ^12^John Hunter Health and Innovation Precinct, New Lambton Heights, NSW, Australia

**Keywords:** telestroke, virtual reality, medical education, stroke workflow, thrombolysis

## Abstract

**Background:**

Telestroke networks aim to address variability in both quality and access to stroke care in rural areas, by providing remote access to expert stroke neurologists. Implementation of telestroke requires adaptation of workflow processes and education. We previously developed virtual reality (VR) workflow training and documented acceptability, utility and feasibility. The effects on acute stroke treatment metrics have not been previously described.

**Aims:**

The overall aim was to improve hyperacute stroke metrics and shorten the time-to-reperfusion therapy administration in rural settings.

**Methods:**

This study applies a natural experiment approach, collecting stroke metric data during transition from a pre-existing pilot to a statewide telestroke service at five rural hospitals. Pre- and post-intervention data included baseline patient demographics and assessment, diagnosis, and treatment delivery metrics. The primary study outcome was door-to-decision time (thrombolysis and endovascular thrombectomy). Secondary outcomes included door-to-computerized tomography time, door-to-thrombolysis time and proportion of patients receiving thrombolysis or thrombectomy treatment. Usage data relating to the VR stroke workflow training of interprofessional healthcare professionals was automatically captured via Wi-Fi. Statistical comparisons of clinical metrics between the pre- and post-intervention time periods, defined as the timeframes before and after VR deployment, were performed.

**Results:**

A total of 2,683 patients were included (April 2013–December 2022); 1910 pre- and 773 post-intervention. All acute stroke time metrics significantly improved post-intervention. The primary outcome, door-to-decision time, decreased from 80 min [56–118] to 54 min [40–76; *P* < 0.001]. Secondary outcomes also improved, including door-to-thrombolysis time (90 min [68–114] vs. 68.5 min [54–90]; *P* < 0.001) and proportion of patients thrombolysed (11 vs. 16%; *P* < 0.001). The proportion of patients transferred for thrombectomy was unchanged (6 vs. 7%; *P* = 0.69). Seventy VR sessions totaling 15 h 39 min of training time were logged. VR training usage varied across sites (3–31 sessions per site).

**Conclusions:**

Delivery of a multi-factorial intervention including infrastructure, funding, education and training (with VR workflow training) as part of a state-wide telestroke rollout was associated with improved acute stroke treatment metrics. Additional work is required to identify the contribution of each intervention component on clinical outcomes and to increase training uptake and sustainment.

## 1 Introduction

There is a disparity in access to best-practice stroke care for patients living in rural areas, both in Australia and internationally (Levi et al., 2019; Hammond et al., 2020). Patients living outside of metropolitan areas are less likely to receive acute reperfusion therapies, leading to worse outcomes after stroke (Edwards et al., 2020; Hammond et al., 2020; Georgakakos et al., 2022). Multiple factors likely contribute to this observation, with limited availability of stroke specialists outside of metropolitan locations likely to be a major factor (Joubert et al., 2008; Prior et al., 2020). This issue is particularly relevant in Australia. Australia is a large country with low population density in rural regions and long distances between comprehensive stroke centers, making timely access to acute stroke therapies across all locations challenging. Approximately 30% of the Australian population lives outside of metropolitan areas (AIHW, 2023), while only 4% of the neurology workforce provide services within these regions and this shortfall is predicted to persist into the future (Simpson-Yap et al., 2023). Compounding this issue, there is a 17% higher risk of stroke in regional and rural Australia (Stroke Foundation, 2020).

To address differences in stroke care access in rural settings, telestroke networks have been developed in Australia (Garcia-Esperon et al., 2022a,b) and worldwide (Silva and Schwamm, 2012; Wilcock et al., 2021), as well as in urban contexts (Cutting et al., 2014). Telestroke pathways aim to provide remote acute specialist support to rural centers, increasing on-site thrombolysis rates and streamlining thrombectomy access (e.g., via coordinated transfer to comprehensive centers). In our state (New South Wales; NSW) a pilot telestroke network was initiated in 2013, which included five rural hospitals. From 2019, this network was expanded via statewide funding to create the NSW Telestroke Service and extended to a total of 23 rural sites supported by a roster of stroke neurologists.

While telestroke networks provide access to stroke neurologist expertise, they still fundamentally rely on the local workforce at regional hospitals for patient management. Integrating telestroke care is not as simple as adding a phone or video call to existing local pathways. Rather, effective implementation requires mechanisms to increase awareness of telestroke processes and staff training in hyperacute stroke therapies and workflow. Changing workflow practices to suit uptake of a telestroke pathway relies on staffing training and education, which is particularly challenging in the Australian rural setting. Staff are geographically distributed, non-specialist, overworked, and transient compared to staff based at metropolitan hospitals (Curran et al., 2006; McLean, 2006; Pond et al., 2009). Less than two-thirds of Australian physicians and nurses involved in stroke management report receiving any interactive or competency-based training (Paul et al., 2019). Further, staff involved in stroke management in small rural centers (< 75 stroke admissions/year) and sites without a dedicated stroke unit are less likely to have opportunities for stroke-specific professional development (Stroke Foundation, 2023).

Our previous local efforts to deliver training to improve rural stroke outcomes highlighted that conventional education alone (e.g., web-based modules) is insufficient to increase access to thrombolysis, suggesting that a more sustained and interactive approach is required (Paul et al., 2014). While intensive quality improvement processes aimed at enhancing team communication and local processes can improve treatment (Meretoja et al., 2012, 2013; Silsby et al., 2019), these interventions are rarely applied in rural settings. Further, limited studies have assessed interventions that apply simulation for stroke workflow training (Tahtali et al., 2017).

Virtual reality (VR) training has emerged as a solution to deliver practical stroke training at rural hospitals with an emphasis on workflow processes. VR can provide immersive, engaging and interactive training, increasing trainee engagement and motivation (Mikropoulos et al., 1998; Jensen and Konradsen, 2018). VR has previously been applied in the context of both medical education and stroke rehabilitation (Laver et al., 2017; McKnight et al., 2020; Piot et al., 2020; Uruthiralingam and Rea, 2020). However, most existing VR training applications targeted at health professionals have only been assessed in small pilot trials and focused specifically on doctors in training and non-physician trainees (Chen et al., 2020; Barteit et al., 2021; Baashar et al., 2022). In stroke, VR studies have primarily targeted patients (e.g., limb rehabilitation), rather than healthcare staff (Laver et al., 2017; Charles et al., 2020; Wiley et al., 2022). To our knowledge the only VR platform providing stroke workflow training for healthcare professionals is TACTICS VR. We initially developed TACTICS VR as part of a package intervention to improve stroke treatment and workflow processes in regional hospitals across three Australian states (Hood et al., 2021; Ryan et al., 2022). The TACTICS VR Stroke Telehealth module was subsequently developed to support rollout of the NSW Telestroke Service by providing a step-by-step walkthrough of workflow processes and awareness of critical elements and infrastructure (Maltby et al., 2023). Our previous work assessing TACTICS VR training yielded promising feedback in terms of acceptability, usability, usefulness, perceived training impact and feasibility. However, previous studies did not assess the effects of VR training on clinical workflow practices or treatment delivery.

The local transition of five rural hospitals from a pilot to a statewide telestroke network provided an opportunity for a “natural experiment.” Natural experiments are increasingly recognized as providing useful data in the context of real-world health interventions, where randomized controlled trials are not practical, feasible or cost-effective (Craig et al., 2017; Crane et al., 2020; Khullar and Jena, 2021). The local circumstances provided a unique and valuable opportunity to assess the impact of the intervention implemented during rollout of a comprehensive statewide telestroke network on stroke metrics.

In the current study, we sought to assess the effects of implementing a statewide telestroke network at five rural sites, as they transitioned from a pilot telestroke service. The intervention was complex and multi-factorial including enhanced training and education (including VR training) and specific infrastructure and funding, as described below. The overall aim was to improve hyperacute stroke metrics and shorten the time-to-reperfusion therapy administration in rural settings. Our primary study outcome was door-to-decision time. Secondary outcomes included the effect of the implementation, including VR training, on (a) door-to-thrombolysis administration time, (b) the proportion of patients receiving thrombolysis treatment, and (c) the proportion of patients transferred for thrombectomy.

## 2 Materials and methods

### 2.1 Telestroke service evolution in New South Wales and study sites

The current study focuses on the implementation of the statewide telestroke service at five rural stroke centers that were initially part of a telestroke pilot project. These sites combined provide stroke services to a population of ~400,000 people.

### 2.2 Clinical workflow changes and intervention

Each site had pre-existing local experience in telestroke-supported acute stroke management and thrombolysis delivery using the same basic model of care, prior to transitioning to the statewide telestroke service. As part of the NSW Telestroke Service implementation, a restructure of the previous pilot service was undertaken, which included an emphasis on enhanced training and education in hyperacute stroke and additional infrastructure and resources. We note that the previous pilot service already included site visits, staff education and regular feedback between stroke neurologists and site coordinators, which were enhanced during the transition to the statewide service. An overview of aspects included in the pilot vs. statewide telestroke service are also provided in Table 1.

**Table 1:** Study sites, relevant dates and overview of relevant components of each telestroke service.

**Study site**	**Pilot telestroke start**	**Statewide initiation**	**Virtual reality deployment**
1	April 2013	September 2021	October 2021
2	June 2014	August 2021	November 2021
3	October 2017	October 2021	May 2021
4	November 2017	March 2020	May 2021
5	November 2017	March 2020	May 2021
**Components of telestroke services**			
**Pilot telestroke (pre-intervention)**		**Statewide telestroke (post-intervention)**	
• Existing stroke team staffing at regional sites		• Funding for additional site coordinator salary at each regional site (1-year)	
• Minimal telestroke neurologist roster at single comprehensive site		• Expanded telestroke neurologist roster across multiple comprehensive hospitals	
• Simplified initial triage informed by FAST		• Triage process supported by web-based ASAP Triage Tool (including NIHSS, mRS, time last seen well)	
• Phone-based neurologist consult		• Phone- and video-based neurologist consult	
• Pre-existing PACS system		• Centralized PACS system	
• Basic education and training package (site visit, *ad-hoc* communication and case feedback)		• Enhanced education and training package (site visit with real-world simulation, weekly virtual audits, virtual reality workflow training, communities of practice)	

FAST, Face/Arms/Speech/Time; NIHSS, National Institute of Health Stroke Scale; mRS, modified Rankin Scale; PACS, Picture Archiving and Communication System.

#### 2.2.1 Infrastructure and funding

Each rural site received 1-year of salary funding for a temporary workforce enhancement embedding a dedicated telestroke project officer (1.0 FTE). Stroke call activation was modified from a positive FAST (Face/Arms/Speech/Time) score (Chen et al., 2022) in the pilot project to an algorithm-based approach using a novel web-based tool (ASAP Triage Tool; Scrawl, Australia), which combines time from stroke onset, baseline National Institute of Health Stroke Scale (NIHSS) scores and baseline modified Rankin Scale (mRS) scores. The interaction between the Emergency Department (ED) teams and the telestroke neurologist were protocolized and homogenized across all the sites. Call format was upgraded from audio-only to video (Microsoft Teams), as previously shown effective in a telestroke context (Meyer et al., 2008), via new dedicated workstation-on-wheels hardware in the rural ED. An initial phone call to the telestroke neurologist was initiated after triage (supported by the ASAP Triage Tool) and before imaging to provide time for the telestroke neurologist to be ready when imaging was available. Subsequent calls are video-based and occur after imaging to support imaging interpretation, treatment decision and patient education. Other aspects included moving to a centralized PACS (Picture Archiving and Communication System) system for image viewing by the telestroke neurologists and improved network transmission for CT imaging transfer. Further, local WiFi capability was audited at the commencement of statewide services and upgrades applied as required.

#### 2.2.2 Education and training

A site visit was conducted with each site prior to commencement on the statewide service, which included a real-world simulation of a telestroke case. Weekly virtual audits of all the patients assessed through the service were scheduled, to which all the rural teams were invited. A major aspect of the training intervention was deployment of TACTICS VR stroke workflow training, which delivered interactive training specifically tailored to the change in clinical workflows associated with transition to the statewide service. A VR headset was deployed to each site, with both pre-existing TACTICS VR workflow training modules installed. Additional detail on the VR-based workflow training is described in detail below.

### 2.3 Study timeframes

Data was included for all patients assessed in each of the five hospitals with stroke-like symptoms throughout the study period, both through the ED or in-hospital stroke activations. The “pre-intervention” data capture period commenced with staggered roll-out of the pilot telestroke service from April 2013 through November 2017 (Table 1). Statewide telestroke initiation commenced progressively across sites from March 2020 to October 2021.

VR training was deployed to each site when it first became available (May 2021) or immediately after each site initiated on the statewide service, whichever was later, from May to November 2021 (Table 1). The “post-intervention” period was defined as the timeframe after each site received access to a VR headset. Data on VR training usage and patient clinical metrics was then collected from each site for a period of at least 12 months until the end of December 2022.

### 2.4 Patient outcome data collection

Clinical data was prospectively collected for all stroke activation cases across the study timeframe and accessible by the comprehensive stroke center throughout the study period. Patient data included baseline demographics, past medical history, baseline NIHSS, brain imaging characteristics (including brain non-contrast computed tomography (CT) findings, vessel status, and CT perfusion core and penumbra volumes), acute reperfusion and therapy decision. The acute brain imaging protocol was the same for all sites and included non-contrast CT, CT angiography (CTA), and CT perfusion (CTP). Either non-contrast CT or magnetic resonance imaging was applied during follow-up at 24–48 h. In the pre-intervention phase, imaging was accessed via screen-sharing between the rural and comprehensive sites. Post-intervention imaging data was available in a statewide PACS repository. All perfusion imaging was post-processed using either MIStar (Apollo Medical Imaging Technology; pilot project) or RAPID (iSchema View, Menlo Park, CA; statewide service) commercial software. Previously validated thresholds were used to calculate CTP ischemic core and penumbra volumes (Bivard et al., 2011). Acute brain imaging was reviewed by the telestroke neurologist on call and transitioned from the use of local imaging servers in the pilot period to an online imaging repository (PACS) developed for the NSW Telestroke Service.

Large vessel occlusion was defined as an occlusion of the proximal segment of the middle cerebral artery (M1 segment), basilar occlusion and/or terminal internal carotid artery with or without extracranial internal carotid (tandem) occlusion. No information regarding M2 occlusions in terms of proximal vs. distal location was available, so this group was not included in the LVO category. Stroke mimic was defined as a combination of: atypical stroke presentation, normal follow-up CT/magnetic resonance imaging, and/or clinically different diagnosis explaining the symptoms given by local team. Door-to-decision time was defined as the time between patient presentation to the ED and when the telestroke neurologist made the decision on most appropriate treatment course [thrombolysis (yes/no) and/or endovascular thrombectomy (yes/no)]. The decision was on an intention-to-treat basis, before obtaining consent. Door-to-needle time was defined as the time between patient presentation to ED and administration of the initial thrombolysis bolus. Rural sites were not thrombectomy-capable and all thrombectomy procedures were performed at the comprehensive center following transport.

### 2.5 VR training application and implementation

**Figure 1:**
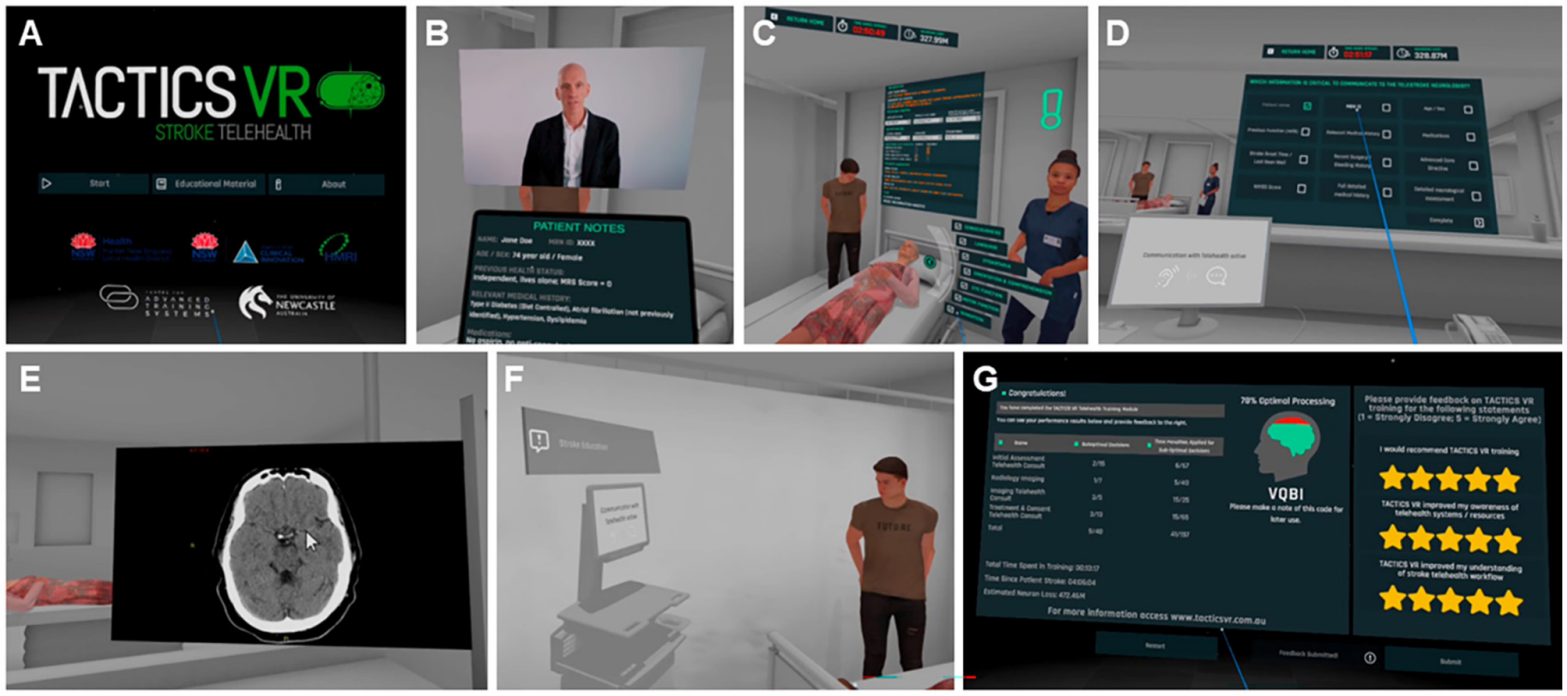
Screenshots and key features of TACTICS VR—Stroke Telehealth. Representative screenshots provided for context in order of presentation in the VR training module, including **(A)** starting scene, **(B)** introduction with talking head video and tablet view, **(C)** patient assessment with representative avatars and interactions, **(D)** initial telestroke neurologist phone call and communication, **(E)** imaging and telestroke neurologist video call supporting interpretation, **(F)** treatment decision and patient education via workstation on wheels, and **(G)** VR user feedback scene. Aspects of the figure are reproduced and adapted from previous publication in *JMIR Serious Games* (Maltby et al., 2023), which was published (and can be reproduced) under the terms of Creative Commons Attribution 4.0 license.

The TACTICS VR workflow training platform includes the initial TACTICS VR—Hyper-Acute Stroke Management and TACTICS VR—Stroke Telehealth modules. Each training module has been previously described in detail (Hood et al., 2021; Ryan et al., 2022; Maltby et al., 2023), including an overview of module design, content inclusions and data capture processes. The respective modules focus on training effective and time-efficient workflow processes for hyper-acute stroke, with each module starting from initial patient notification and progressing through assessment, imaging, decision, treatment and transport (Hood et al., 2021; Ryan et al., 2022; Maltby et al., 2023). The intent in the current study was for staff to use the TACTICS VR—Stroke Telehealth module as it specifically focuses on telestroke workflow processes. Access was also provided to the 1st TACTICS VR—Hyper-Acute Stroke Management module, as sites previously used the application during the initial TACTICS clinical trial (52 completed VR sessions between November 2019 and March 2020; Hood et al., 2021; Ryan et al., 2022). As training sessions were non-identifiable, it is not possible to determine how many of the staff that previously completed VR training in the TACTICS trial remain at the sites in the current intervention period. The training modules provides an interactive, first-person virtual walkthrough of a single stroke case from the physician's perspective with emphasis on workflow processes (Figure 1). Users are initially oriented to the VR environment and introduced to the stroke and neuron clock (a gamification element that emphasizes the importance of timely decision-making and negative consequences of suboptimal decisions) and the virtual tablet (which displays collected patient information). Gamification has been extensively documented to support trainee engagement and learning in a range of settings (Caponetto et al., 2014). Workflow proceeds via a linear narrative from an opening title screen/introduction, followed by trainee demographics capture, pre-notification/initial assessment, CT imaging, consent, on-site thrombolysis, patient transfer for endovascular thrombectomy and user performance feedback. Content is delivered via an interactive interface, to promote active learning and trainee engagement and user feedback. For both TACTICS VR modules, simple user demographics (hospital, previous TACTICS VR usage) and responses for all user decisions are automatically transmitted to a password-protected, cloud-based reporting database to support research and implementation. Each individual training session (i.e., one user completing one virtual workflow process) was logged and metrics are reported (e.g., session time, demographics, and errors). The TACTICS VR—Stroke Telehealth module also incorporates additional demographic data collection (department, position title, stroke management experience by patient numbers, and years of service) and logs incomplete sessions. Incomplete sessions are logged when a user initiates training and completes the initial demographics responses but does not complete training through to the final feedback screen.

A stand-alone Meta Quest 2 headset (Meta previously Oculus, Menlo Park, CA, USA) was provided to each hospital site, with enterprise management software supporting remote hardware monitoring. A mobile WiFi router was also provided, and no issues with signal bandwidth or connectivity were noted or reported. The TACTICS VR—Hyper-Acute Stroke Management was upgraded from the previously published version, which was previously deployed on the Oculus Go headset. Telestroke site coordinators were initially briefed and trained with the VR headset on a site-by-site basis via web-conferencing and provided supporting documents (e.g., quick access guide). Site coordinators were requested to encourage relevant staff at their hospital to use VR training. Clinical staff were then free to use the VR training module on-site on their own time. Additional support was offered on a needs-based basis but was not requested by any site.

### 2.6 Statistical analysis

Descriptive statistics are presented as count (%), mean (standard deviation; SD) and median (interquartile range; IQR). Variables of age, baseline NIHSS, gender, and pre-morbid mRS were compared using chi-squared test for categorical variables and ANOVA (mean)/Kruskal-Wallis (median) for continuous variables. The presented metrics were not adjusted based on hospital or patient baseline characteristics. An alpha level of 0.05 was specified for all tests and confidence intervals. The data were analyzed in SAS v9.4.

### 2.7 Ethics statement

Ethics approval for NSW Telestroke Service data collection and reporting was approved by the Prince of Wales Hospital Human Research Ethics Committee. TACTICS VR training implementation was approved by the Hunter New England Health Human Research Ethics Committee (REGIS Refs. 2019/ETH01238 and 2019/ETH13062), lodged with the University of Newcastle Human Research Ethics Committee (H-2019-0343) and by Hunter New England Health Human Research Ethics Committee exemption (#AU202208-13). All study sites provided consent to participate.

## 3 Results

### 3.1 Clinical outcomes

#### 3.1.1 Patient characteristics

A total of 2,683 patients were included in the current analysis: 1910 in the pre-intervention and 773 in the post-intervention period (Table 2). The median age was 73 [63–82] years, with patients in the pre-intervention period slightly younger than those in the post-intervention period (73 [62–81] vs. 74 [63–83] years; *P* = 0.03). Patients in the pre-intervention period had a slightly milder stroke presentation, with baseline NIHSS score of 4 [2–10] compared to 5 [3–11] in the post-intervention period (*P* < 0.001). The median baseline mRS was 0 [0–2] in both periods. Patients assessed in the pre-intervention period were less likely to have a past medical history of hypercholesterolemia (29 vs. 41%, *P* < 0.001), with otherwise similar risk factors. The proportion of patients on antiplatelet or anticoagulant therapy was similar across both time periods. A total of 1,065 patients (56%) had a final diagnosis of ischemic stroke in the pre-intervention period, similar to the 452 patients (58%) in the post-intervention period (*P* = 0.2). A total of 460 patients (24%) were classified as stroke mimics in the pre-intervention period, compared to 159 patients (21%) in the post-intervention period (*P* = 0.05). We are unaware of any mimic cases receiving tPA treatment in the current study. A greater proportion of intracerebral hemorrhage was observed post-intervention (10%) compared to pre-intervention (6%; *P* < 0.001). Among all patients with a confirmed ischemic stroke, the mean baseline brain volume identified as hypoperfused via CTP imaging was smaller in patients assessed in the pre-intervention period 33.4 (59.3) mL compared to the post-intervention period 96.9 (97.4) mL (*P* < 0.001). Furthermore, patients in the pre-intervention period were less likely to have a visible vessel occlusion (40 vs. 52%; *P* < 0.001) or a large vessel occlusion (19 vs. 23%; *P* = 0.04). Mean core and penumbra volumes was lower in pre-intervention compared to post-intervention [8.4 (22.0) mL vs. 21.6 (41.2) mL and 25 (44.9) mL vs. 75.1 (76.7) mL, respectively; *P* < 0.001]. It is unclear what specifically underlies this difference, but it may relate to changes in imaging analysis software applied in the pre- vs. post-intervention period and/or reflect improvements in patient identification and triage enriching for the patient subset with vessel occlusion.

**Table 2:** Baseline patient characteristics and final diagnosis in the total study population and pre- vs. post-intervention periods.

	**Total population *n* = 2,683**	**Intervention period**		
		**Pre-intervention**, ***n*** = **1,910**	**Post-intervention**, ***n*** = **773**	***P*** **-value**
Median age, y [IQR] (*n* = 2,674)	73 [63–82]	73 [62–81]	74 [63–83]	0.03
Male sex, *n* (%) (*n* = 2,669)	1,462 (55)	1,056 (56)	406 (53)	0.21
Median baseline NIHSS score [IQR] (*n* = 2,608)	4 [2–10]	4 [2–9]	5 [3–11]	< 0.001
Baseline mRS 0–3, *n* (%) (*n* = 2,586)	2,512 (97)	1,802 (97)	710 (98)	0.02
Median baseline mRS [IQR] (*n* = 2,586)	0 [0–2]	0 [0–2]	0 [0–2]	0.07
**Clinical history**, ***n*** **(%)**				
Hypertension (*n* = 2,677)	1,454 (54)	1,025 (54)	429 (55)	0.43
Hypercholesterolemia (*n* = 2,678)	863 (32)	547 (29)	316 (41)	< 0.001
Prior stroke or transient ischemic attack (*n* = 2,678)	663 (25)	472 (25)	191 (25)	0.97
**Baseline medication**, ***n*** **(%)**				
Antiplatelets (*n* = 2,678)	760 (28)	521 (27)	239 (31)	0.06
Anticoagulation (*n* = 2,678)	445 (17)	301 (16)	144 (19)	0.07
**Baseline imaging characteristics** ^*^				
Mean core volume, mL (SD) (*n* = 1,499)	10.8 (27.0)	8.4 (22.0)	21.6 (41.2)	< 0.001
Mean penumbra volume, mL (SD) (*n* = 1,499)	34.3 (55.7)	25 (44.9)	75.1 (76.7)	< 0.001
Mean hypoperfused (core + penumbra) volume, mL (SD) (*n* = 1,497)	45.1 (72.2)	33.4 (59.3)	96.9 (97.4)	< 0.001
Visible vessel occlusion, *n* (%) (*n* = 1,517)	663 (44)	426 (40)	237 (52)	< 0.001
Large vessel occlusion, *n* (%) (*n* = 1,517)	306 (20)	200 (19)	106 (23)	0.04
**Final diagnosis**, ***n*** **(%)**				
Ischemic stroke	1,517 (57)	1,065 (56)	452 (58)	0.20
Transient ischemic attack	223 (8)	169 (9)	54 (7)	0.11
Intracerebral hemorrhage	181 (7)	106 (6)	75 (10)	< 0.001
Stroke mimic	619 (23)	460 (24)	159 (21)	0.05
Undetermined diagnosis	58 (2)	26 (1)	32 (4)	< 0.001

^*^In the group of confirmed ischemic stroke patients (n = 1,517).

mRS, modified Rankin Score; NIHSS, National Institutes of Health Stroke Scale.

#### 3.1.2 Acute stroke metrics and reperfusion therapy decisions

All acute stroke workflow time metrics assessed improved in the post-intervention period compared to the pre-intervention period (Table 3). The median time from ED arrival to telestroke neurologist call decreased from 35 [20–63] min pre-intervention to 20 [12–33] min post-intervention (*P* < 0.001). Median time from patient ED arrival to onset of brain imaging decreased from 53 [32–90] min to 37 [25–55] min (*P* < 0.001). Further, door-to-decision time decreased from 80 min [56–118] to 54 [40–76] min (*P* < 0.001). In the subset of patients with confirmed ischemic stroke as a final diagnosis, changes in door-to-decision time were similarly decreased (pre-intervention = 80 min [56–115]; post-intervention = 55 min [40–79]; *P* < 0.001). The time from last seen well to ED arrival increased from the pre-intervention period from 132 [76–395.5] min to 167 [87–452] post-intervention (*P* = 0.002).

**Table 3:** Acute stroke management, times and treatment.

	**Total population**	**Intervention period**		
		**Pre-intervention**	**Post-intervention**	***P*** **-value**
Last time seen well to ED (rural) arrival, median [IQR] (*n* = 2,210)	140 [79–415]	132 [76–395.5]	167 [87–452]	0.002
Time from ED arrival (rural) to telestroke neurologist call, median [IQR] (*n* = 2,110)	29 [17–53]	35 [20–63]	20 [12–33]	< 0.001
Time from ED arrival (rural) to onset of brain imaging, median [IQR] (*n* = 2,286)	47 [29–80]	53 [32–90]	37 [25–55]	< 0.001
Time from ED arrival (rural) to treatment decision, median [IQR] (*n* = 2,223)	70 [49–106]	80 [56–118]	54 [40–76]	< 0.001
Time from ED arrival (rural) to thrombolysis bolus, median [IQR] (*n* = 297)	81 [62–105]	90 [68–114]	68.5 [54–90]	< 0.001
Proportion patients ≤ 60-min from ED arrival (rural) to thrombolysis bolus, no (%) (*n* = 297)	67 (23)	26 (15)	41 (34)	< 0.001
**Reperfusion therapies**, ***n*** **(%)**				
Intravenous thrombolysis	340 (13)	214 (11)	126 (16)	< 0.001
Endovascular thrombectomy	169 (6)	118 (6)	51 (7)	0.69

ED, Emergency department.

Time metrics reported in minutes.

A total of 340 patients (13%) were treated with thrombolysis at the rural sites across the entire study. There was a significant increase in the proportion of patients thrombolysed in the post-intervention period (126 patients, 16%) compared to the pre-intervention period (214, 11%; *P* < 0.001). Among the 297 patients treated with thrombolysis with complete time metrics data, the door-to-thrombolysis delivery (door-to-needle time) decreased from 90 min [68–114] to 68.5 min [54–90] (*P* < 0.001). This corresponded with 15% of patients with door-to-thrombolysis delivery times within the benchmark of 60-min in the pre-intervention period, which increased to 34% post-intervention (*P* < 0.001). A total of 169 patients (6%) were transferred for endovascular thrombectomy during the study period, with no significant difference in the proportion pre- vs. post-intervention (6 vs. 7%, respectively, *P* = 0.69).

### 3.2 TACTICS VR training usage

A total of 70 VR training sessions were logged across all sites between May 2021 and December 2022. This included 51 sessions with TACTICS VR—Stroke Telehealth (24 completed and 27 incomplete sessions) and 19 complete sessions with TACTICS VR—Hyper-Acute Stroke Management. The number of logged training sessions per site ranged from 3 to 31.

VR training time in completed sessions totaled 15 h, 39 min for both modules, including 6 h 43 min with TACTICS VR—Stroke Telehealth and 8 h 56 min with TACTICS VR—Hyper-Acute Stroke Management. Mean VR session times were 16 min 48 s ± 3 min 23 s (mean ± SD) for TACTICS VR—Stroke Telehealth and 28 min 13 s ± 22 min 30 s for TACTICS VR—Hyper-Acute Stroke Management. User response accuracy was 83 ± 9% for TACTICS VR—Stroke Telehealth and 71 ± 9% for TACTICS VR—Hyper-Acute Stroke Management. Training penalizes sub-optimal decisions and responses with time penalties, resulting in an average of 26 ± 19 min and 48 ± 32 min per session of “time penalties” applied per session, respectively.

Training usage was highest directly after VR deployment, with 50% of completed TACTICS VR—Stroke Telehealth sessions within the 1st month (12/24) and 75% in the first 4 months (18/24). Similarly, 53% of completed TACTICS VR—Hyper-Acute Stroke Management sessions occurred in the 1st month (10/19) and 84% in the first 4 months (16/19).

User responses to demographic questions were captured in-headset for TACTICS VR—Stroke Telehealth sessions only. Users primarily worked in Emergency Care (*n* = 18, 75% of sessions), followed by Acute Stroke/Neurology (*n* = 2, 8%), Radiology (*n* = 1, 4%), Intensive Care (*n* = 1, 4%), and Other (*n* = 2, 8%). Users were employed as Nurses (*n* = 11, 46%) or Doctors (including “in training,” *n* = 5, 21%; staff specialist, *n* = 4, 17%), with the remainder Radiographers (*n* = 1, 4%) or Other (*n* = 2, 8%). Approximately 42% had previously used TACTICS VR training (*n* = 10/24), likely as part of the TACTICS trial in 2019 (Hood et al., 2021; Ryan et al., 2022). Users had relatively limited previous stroke management experience in terms of both total patient numbers and years, 33% of them had managed < 10 stroke patients in their career and 33% had 5 or fewer years of experience in stroke management (8/24; 5 = 0 years, 3 = 1–5 years). In contrast, 25% of users (6/24) had previously managed >40 stroke patients and 8% (2/24) had over 15 years of experience.

## 4 Discussion

We assessed the effect of transitioning to a state-wide telestroke service on acute stroke metrics and treatment rates at five rural hospitals. All sites transitioned from a previous pilot telestroke service, with incorporation of education and training activities (including dedicated VR-based workflow training) and additional infrastructure and funding. The primary study outcome, door-to-decision time for all patients, decreased by 26 min from 80-to 54-min. Similar results were found in the time-to-thrombolysis delivery for those patients receiving thrombolysis treatment, decreasing from 90-min pre-intervention to 68.5-min post-intervention. Further, the number of patients treated with thrombolysis increased (to 16% post-intervention from 11% pre-intervention). No change was identified in the proportion of patients transferred for endovascular thrombectomy. VR training was feasible, with 70 training sessions logged across both TACTICS VR modules representing over 15-h of training time, although uptake was lower than expected.

Stroke treatment times and thrombolysis rates markedly improved in the post-intervention period. By comparison, the Australian National Stroke Audit reported slightly worsened metrics across part of the study period, between 2017 (Stroke Foundation, 2017) and 2023 (Stroke Foundation, 2023). No national or state-level data were available for the rest of the study period. For the two metrics that overlapped with our analysis, national thrombolysis rates decreased from 13 to 10% and the proportion of patients with door-to-thrombolysis delivery times < 60-min decreased from 30 to 29% from 2017 to 2023, respectively. Metrics in the pre-intervention period of the current study were comparable with the national audit metrics (11% thrombolysis rate; 15% ≤ 60-min timeframe). In contrast, post-intervention metrics markedly improved to 16% thrombolysis rate and 34% door-to-thrombolysis time ≤ 60-min. This supports the conclusion that the observed improvements relate to the study intervention and are unlikely to reflect broader trends of improved stroke management during the study period.

Study design constraints prevented us from performing causal analyses to determine the effects of individual intervention aspects on treatment metrics. Further, numerous confounding factors between hospitals (e.g., size, staffing, and resources) prevented a meaningful analysis comparing individual site data (e.g., correlating VR usage by site outcomes). We noted that VR usage was variable across sites and overall uptake was lower than expected with the majority of usage occurring in the first 4-months after deployment, consistent with our previous studies (Hood et al., 2021; Ryan et al., 2022; Maltby et al., 2023). As reported in our previous studies (Hood et al., 2021; Ryan et al., 2022; Maltby et al., 2023), we speculate that limited uptake may relate to local staffing, time availability for training, familiarity and attitudes to new technology and communications. Funding to support on-site stroke coordinators and communications (e.g., initial site visit, community of practice, and case review) likely contributed to broader staff up-skilling and improved processes, although this could not be objectively quantified. Anecdotally there was strong uptake of the web-based ASAP Triage Tool to streamline triage and stroke assessment but objective usage metrics were not captured. Further, research team members noted that the improved telehealth technology and PACS system reduced technical issues and improved overall communication. Imaging interpretation software changes may have had an effect on patient assessment metrics, as RAPID assessment has previously been shown to provide larger estimates of perfusion lesion volume compared to MIStar (Bivard et al., 2022). While an increase in time last seen well-to-ED arrival time was observed, we note that the current intervention did not address the pre-hospital period and the criteria for telestroke activation remained unchanged. As such, it is unlikely that this observed difference directly affected reported telestroke time metrics in this study but may reflect unrelated changes in pre-hospital stroke triage and transport decision-making across the study period. Future studies are now needed to determine the specific impact of each intervention.

Understaffing and high staffing turnover are chronic issues in EDs across rural Australia. In high pressure scenarios and under workload/resourcing stresses, education activities may be sacrificed because of the overwhelming competing demands of patient care delivery. Further, the shift-working nature of ED staff prevents capturing a large proportion of staff in scheduled education sessions. We previously demonstrated that deployment of conventional education in isolation is insufficient to improve thrombolysis metrics (Paul et al., 2014). To address this challenge, we deployed multiple training interventions using multiple modalities. VR training was specifically developed to provide flexibility, with training available at any time on-site. VR technology allows expert advice to be embedded within the headset and reduces travel requirements and logistics, compared to traditional training delivered face-to-face by a stroke neurologist. VR has been assessed for medical training in previous studies (McKnight et al., 2020; Piot et al., 2020; Uruthiralingam and Rea, 2020), with consistent findings VR improves trainee engagement (Mikropoulos et al., 1998; Jensen and Konradsen, 2018) and skill acquisition (Roberts et al., 2017; Bouaicha et al., 2020). Furthermore, the use of VR for stroke workflow training proved to be a useful platform during the coronavirus pandemic (Maltby et al., 2023), where face-to-face interactions were minimized. The initial site visit provided an opportunity for team-based real-world simulation. Additional *ad hoc* training was provided via virtual patient audits, which all sites were invited to attend. Participation at site visits and virtual audits was limited to the staff available at the designated times and attendance was not logged. By applying a multi-factorial approach, intent was to provide multiple avenues for staff education and training to improve stroke metrics.

Local circumstances during the transition from an initial pilot telestroke network to the statewide NSW Telestroke Service, provided a unique opportunity for a “natural experiment.” While natural experiments allow for assessment of real-world implementation where randomized controlled trials are not possible (Craig et al., 2017; Crane et al., 2020; Khullar and Jena, 2021), study constraints limit the ability to determine causal relationships, compared to randomized controlled trial design and we are unable to demonstrate a causative effect of VR training on acute stroke metrics. Causal inference can be supported through collection of additional qualitative data (Craig et al., 2017). To this end, the findings in the current study are supported and consistent with our previous reports, which included user feedback and indicated VR training is acceptable, feasible and has perceived training impact in both clinical trial and telestroke contexts (Hood et al., 2021; Ryan et al., 2022; Maltby et al., 2023). An analysis of the effect of a package intervention including the first TACTICS VR training module on clinical processes and stroke treatment metrics in a stepped-wedge clinical trial context is also currently underway (Ryan et al., 2022). While we did not report qualitative data in the current study, our previous research has highlighted barriers to VR training uptake including staffing, existing attitudes to training, local processes and competing priorities (Hood et al., 2021; Ryan et al., 2022; Maltby et al., 2023).

We highlight several key strengths of the current study including: data collection within a real-world clinical context; comprehensive capture of clinical metrics data over >9-years including >2,600 patients; inclusion of multiple rural hospitals with varying size, remoteness and pre-existing training support; delivery of a complex and multi-faceted intervention strategy addressing education and training, staffing and infrastructure; inclusion of interactive VR workflow training with immediate, anonymous, and automated data capture.

There are also several limitations that should be considered when interpreting the study findings and applying the learnings to other contexts. Firstly, VR education was implemented as part of a broader education and training approach within the context of a statewide telestroke network rollout to sites that previously enrolled in a pilot telestroke project. There was also no capacity to link data for individual clinician completion of VR training and individual patient outcomes. Further, we were unable to assess for associations between VR usage and patient outcomes by site. As a result, study constraints prevented us from establishing causation or determining specific effects of each intervention component, as discussed above. Secondly, statewide telestroke initiation and VR deployment dates differed for each site, with gaps ranging from 1 to 14-months. This resulted as the VR module was not available until May 2021 and unanticipated delays altered “go live” dates, in part due to COVID-19 restrictions overlapping the study timeframe. Thirdly, VR uptake was limited (70 total sessions including 51 with TACTICS VR—Stroke Telehealth and 19 sessions with the 1st TACTICS VR module). Further, most VR training occurred in the first 4-months after deployment (75%; 18/24 and 84%; 16/19 for each module, respectively) and subsequently declined over the study period. Engagement with the other training components was not specifically tracked or logged to assess uptake. We propose there is potential for optimization to increase uptake and support long-term sustainment, which should be informed by the implementation science literature (Michie et al., 2011; Atkins et al., 2017). For example, we hypothesize that integration of an embedded “VR champion” may increase sustainment and alternative delivery approaches may optimize uptake (e.g., facilitated workshops and communications). Fourthly, our results are limited to a single geographical region in Australia, which might limit generalizability. Further, analysis was not adjusted for hospital- or patient-based characteristics due to the multitude of potential factors to consider and limitations of the “natural experiment” design (Dunning, 2008). It may be necessary to consider local resourcing, hospital characteristics and patient populations when implementing similar interventions in other contexts. Fifthly, due to the complexity and real-world design of the current study, no control comparator group was available. As a result, all analysis has been performed comparing pre- vs. post-intervention metrics. Finally, much of the study timeframe overlapped the ongoing COVID-19 pandemic, particularly the period corresponding with the transition to the statewide network (early 2020 into 2021). The impact of COVID-19 may have independently altered ED workflow and education processes, although we would anticipate that changes would have resulted in increased stroke treatment time metrics and decreased education activities.

## 5 Conclusion

In conclusion, transition from a pilot to a statewide telestroke service, which included education and training activities and additional infrastructure and funding, was associated with improved acute stroke metrics. This effect is likely multi-factorial, due to the effects of multiple components of the complex intervention. The current study was not designed to determine the causal effect of individual intervention components on stroke outcomes. We propose that VR training can be a useful tool to improve education and clinical care in real-world contexts for stroke, in additional settings relevant to stroke (e.g., pre-hospital paramedic training) and other hyperacute indications (e.g., trauma and cardiac). Future studies will be useful to determine the relative effects of each specific intervention on outcomes and to assess approaches to increase training uptake and improve long-term sustainment.

## Data availability statement

The raw data supporting the conclusions of this article will be made available by the authors, without undue reservation.

## Ethics statement

The studies involving humans were approved by Prince of Wales Hospital Human Research Ethics Committee and Hunter New England Health Human Research Ethics Committee. The studies were conducted in accordance with the local legislation and institutional requirements. The ethics committee/institutional review board waived the requirement of written informed consent for participation from the participants or the participants' legal guardians/next of kin because of the nature of the hyper-acute medical emergency associated with stroke.

## Author contributions

CG-E: Conceptualization, Formal analysis, Investigation, Methodology, Visualization, Writing – original draft, Writing – review & editing, Funding acquisition, Supervision. SM: Conceptualization, Data curation, Formal analysis, Investigation, Methodology, Visualization, Writing – original draft, Writing – review & editing. KB: Conceptualization, Investigation, Methodology, Supervision, Writing – review & editing, Funding acquisition. MH: Data curation, Formal analysis, Investigation, Methodology, Visualization, Writing – review & editing. BC: Investigation, Writing – review & editing. WO'B: Investigation, Methodology, Supervision, Writing – review & editing. JE: Investigation, Methodology, Supervision, Writing – review & editing. TA: Investigation, Writing – review & editing. LE: Investigation, Writing – review & editing. CB: Investigation, Writing – review & editing. CD: Investigation, Writing – review & editing. MP: Investigation, Supervision, Writing – review & editing. FM: Investigation, Supervision, Writing – review & editing. JD: Formal analysis, Investigation, Writing – review & editing. DL: Formal analysis, Investigation, Writing – review & editing. DB: Formal analysis, Investigation, Writing – review & editing. MK: Conceptualization, Investigation, Writing – review & editing. JW: Supervision, Writing – review & editing. CL: Investigation, Writing – review & editing, Funding acquisition, Methodology, Supervision. NS: Conceptualization, Investigation, Methodology, Project administration, Supervision, Writing – original draft, Writing – review & editing. FW: Conceptualization, Funding acquisition, Investigation, Methodology, Project administration, Supervision, Writing – original draft, Writing – review & editing.

## Virtual reality NSW telestroke group contributors

- Chris Oldmeadow
- Rachel Peake
- Jaclyn Birnie
- Amanda Buzio
- Jennifer Steel
- Kim Parrey
- Emma McCartney
- Thembelihle Mathe
- Matthew Shepherd
- Lisa Dark
- James Hughes
- Kate Jackson
- Claire Gill
- Courtney Dixon
- Skye Russell
- Natalie Wilson
- ED Directors/leads and contributing team members
- Teams involved in Imaging/IT support.
